# Supplementary material for: Rapid label-free analysis of Opisthorchis viverrini eggs in fecal specimens using confocal Raman spectroscopy
Source: PLoS One. 2019 Dec 26;14(12):e0226762. doi: 10.1371/journal.pone.0226762 (PMC6932817; doi:10.1371/journal.pone.0226762)
Supplement: Attachment — Submitted filename: Point by point Responses to Reviewers.docx [file pone.0226762.s001.docx]

Dear Editor

Thank you for reviewing our manuscript. We appreciate the careful review, comments, and helpful suggestions of you and the reviewers. Please find attached a revised version of our manuscript, “Rapid label-free identification of *Opisthorchis viverrini* eggs in fecal specimens using confocal Raman spectroscopy,” in submission to PLOS ONE. In it, we have responded, point-by-point, to the comments and suggestions regarding our initial submission, and believe that the manuscript is improved as a consequence. In particular, we have revised the manuscript as follows.

**Journal requirements:**

1. Please ensure that your manuscript meets PLOS ONE's style requirements, including those for file naming. The PLOS ONE style templates can be found at <http://www.journals.plos.org/plosone/s/file?id=wjVg/PLOSOne_formatting_sample_main_body.pdf> and <http://www.journals.plos.org/plosone/s/file?id=ba62/PLOSOne_formatting_sample_title_authors_affiliations.pdf>

Reply: we have checked and modified the manuscript to meet the style requirements of PLOS ONE.

2. In your Methods section, please provide additional location information of the collection sites of the fish, including geographic coordinates for the data set if available.

Reply: Per your request, we have provided more location information about the collection site in the revised manuscript (lines 71-73).

3. In your Methods section, please provide additional information regarding the permits you obtained for the work. Please ensure you have included the full name of the authority that approved the field site access and, if no permits were required, a brief statement explaining why.

Reply: The fish used in this study were purchased from a local fish market; thus, no specific permission was required. Please note that we added the sentence, “No specific permission was required for sampling fishes in public locations.” In the Materials and Methods section (line 73).

4. To comply with PLOS ONE submissions requirements, please provide methods of sacrifice of the fish in the Methods section of your manuscript.

Reply: No humane endpoint was used in this study because we used only dead fish purchased from a local fish market. Please note that we added the sentence, “No humane endpoint was used because only dead fish from a food market were used” (lines77). After obtaining them, the dead fish were processed by the methods outlined in lines 74-81in the Materials and Methods section.

5. In your Methods section, please include a comment about the state of the hamsters following this research. Were they euthanized or housed for use in further research? If any animals were sacrificed by the authors, please include the method of euthanasia and describe any efforts that were undertaken to reduce animal suffering.

Reply: No euthanized and killed animals were used - only the fecal sediment containing *O. viverrini* eggs was collected. After this study, the hamsters were housed for eggs collection of other studies until the animals are expired. Please see revised manuscript, lines 94-96. Please note that the study protocol was approved by the Animal Ethics Committee of Khon Kaen University (AEMDKKU 029/2018) and animal care was carried out in strict accordance with the recommendations laid out in the National Research Council of Thailand's Guide for the Care and Use of Laboratory Animals. The experiment was made to minimize pain and suffering to the animals and only fecal specimens were collected. Five male hamsters were housed under conventional conditions (12 hours dark-light cycles, temperature 25 ± 2°C), fed with a stock diet (Smart heart, Thailand) and given filtered bottled water ad libitum. The hamster health was observed daily and cage bedding changed twice a week. None of experimental hamsters showed serious illness or severe health problems during the study.

**Additional Editor Comments:**

The MS by Chuchuen and others presented an interesting alternative for the diagnosis of Opisthorchis eggs, an important task in endemic areas using the microscopy to differentiate from others common trematodes occurring in the same region. However, there are some points to be clarified and important suggestions by the reviewers which are critical to assure the quality of the results and to improve the quality of the study; improving the controls of the experiments, add a blind test comparison by different microscopists, and use samples from human naturally infected. Also, I may suggest a revision on the usage of certain terminology eg.: in the lInes 160-161 it is stated the profile of the eggs are "unique", but there are no tests shown using others helminthes eggs and protozoa oocists to make this statement, also "in situ" or "noninvasive" (line 183 and in other passages) should be carefully checked.

Reply: The objective of this study was to investigate the feasibility of Raman spectroscopy for rapid, label-free detection of liver fluke eggs in fecal specimens. As a proof-of-concept study, *O. viverrini* eggs obtained from hamster and human feces were chosen as a reprentative group of liver fluke eggs to demonstrate the promising capability of Raman spectroscopy for this application. Our goal was to report a set of Raman marker bands of *O. viverrini* eggs that could be used to identify the eggs in feces by the Raman spectroscopic technique. Thus, the control of this study consisted of the Raman spectra of the fecal debris around *O. viverrini* eggs. Results confirmed that the Raman bands of *O. viverrini* eggs found were different from those of the surrounding fecal debris (see Fig 5). Clearly, the analysis of others helminthes eggs and protozoa oocysts is very useful and will be an interesting topic for our follow up studies.

This study was conducted in a blind manner. The specimens were observed by two groups of observers. One group consisted of parasitologists who prepared and collected *O. viverrini* eggs, while the other group consisted of Raman microscopists who ran the Raman spectroscopic experiments and investigated the Raman spectra.

We appreciate your comments about using human naturally infected *O. viverrini* eggs in the study. As per your suggestion, we acquired *O. viverrini* eggs from fecal specimens of four patients and ran additional Raman experiments to obtain Raman spectral data of those human *O. viverrini* eggs. The results are now presented in the new Figures 3 and 4 and lines 142-146in the Results section. The peak locations of the Raman spectral bands of human *O. viverrini* eggs were included in the revised Table 1. Please note that the methods of human specimen acquisition and preparation were included in lines 102 to 109 in the Materials and Methods section.

As for your additional comments, we have deleted the word “unique” and revised the statement. This revision can be seen in the revised manuscript. In addition, we realized that the use of “in situ” and “noninvasive” might not be suitable for our contexts of work here. We deleted the word “in situ” from the entire manuscript. As for the “noninvasive” word, we realize that we used both “non-invasive” and label-free”, making it too redundant. In fact, “label-free” would be a more suitable word and sufficient to define our technique here. Therefore, we deleted “noninvasive” throughout the manuscript, but have remained to use “label-free” to describe our methodology.

**Reviewer #1:**

Authors’ purposes, in this manuscript, is to “develop a label-free methodology for in situ identification of O. viverrini eggs using non-invasive Raman spectroscopy”. The manuscript seems to me well written, well riding. However, I am not an English native for a good language revision.

Reply: Thank you very much for the positive comments.

Global comments: Authors have choice an interesting, relevant and pertinent diagnosis suggestion. They need to be stimulated to go on working on the field. However, in my point of view, manuscript evidences three main concerns:

1) Lack of information related with the diagnostic value of faecal microscopic examination and its accuracy (specificity and sensitivity; false negatives? etc.);

Reply: The objective of this proof-of-concept study was to demonstrate the promising capability of Raman spectroscopy for label-free identification of *O. viverrini* eggs in hamster and human feces. Clearly, follow-up studies are needed to observe the diagnostic value and accuracy of our Raman spectroscopic technique vs. the traditional faecal microscopic examination for identification of different types of parasitic eggs.

2) No data related with marker bands for Clonorchis sinensis and other eggs of Opisthorchis’s derived from Raman spectroscopy application

Reply: Our goal was to demonstrate that Raman spectroscopy is a promising technique for detecting liver fluke eggs. Eggs of *O. viverrini* were chosen as a representative group of liver fluke eggs to develop our technique and demonstrate our proof-of-principle methodology. Clearly, the Raman spectroscopic analysis of Clonorchis sinensis and other eggs of Opisthorchis is very useful, and will be a topic for our follow up analysis.

3) Finally, this manuscript describes results obtained with faecal samples of experimental infection in Hamsters. Why authors do not use faecal human samples? Validate their results with human samples seems to me crucial.

Reply: We acquired *O. viverrini* eggs from fecal specimens of 4 human subjects and conducted a new set of additional experiments to acquire Raman spectra of those human-derived *O. viverrini* egg. The results are now presented in the new Figures 3-4 and the revised Table 1 (lines 142-146). The new results demonstrated close similarity between the Raman spectral bands of *O. viverrini* eggs derived from hamster and human, confirming that this methodology can be applied to detect *O. viverrini* eggs in human feces.

Specific comments: The manuscript describes an innovative and interesting technique. In My Point Of View, now, the manuscript is not in good shape for publication.

Reply: We appreciate the very helpful comments of the reviewer. We have responded to the suggestions for revisions of our original manuscript, and believe that the revised version of the manuscript has satisfactorily addressed them and, in so doing, has provided a more complete presentation of our study.

**Reviewer #2:**

This manuscript entitled Rapid label-free identification of Opisthorchis viverrini eggs in fecal specimens using confocal Raman spectroscopy presents an originality to apply new technology to solve the problem in differential identification of Opisthorchis viverrini-, Clonorchis sinensis-, O. felineus- and intestinal minutes eggs in fecal sample. This will be rapid and really useful for assessment of actual epidemiology, prevention and control.

However, there are still the major points that need to be addressed by the authors regarding the comments below.
 The major concerning:

1. Authors need to include other opisthorchiidae eggs, C. sinensis and O. felineus, and species confirm intestinal minutes in this manuscript. As mentioned by the authors that “The averaged Raman spectra of 10 O. viverrini eggs from each infected hamster showed little variability, revealing three unique Raman bands specific to O. viverrini eggs.” (L130-132), it is too early because there is no the spectrum of other opisthorchiidae and intestinal minutes eggs to confirm.

Reply: We appreciate that the reviewer pointed this to us. We realized that the original statement was misleading. Therefore, instead of saying that the three Raman bands were unique to *O. viverrini* eggs, we revised the statement to say that the *O. viverrini* eggs consisted of three Raman bands. The revised statement can be seen in lines 140-142in the revised manuscript. We agree that it is too early to conclude that these bands are unique to O. viverrini eggs since there are no data from other eggs shown in this manuscript. Again, we note that our intent for this proof-of-principle study has been to demonstrate application of our new methodology based on Raman spectroscopy by applying it to detect a type of fluke egg, *O. viverrini*. Currently, we are applying our developed methodology to measure other opisthorchiidae eggs and minute intestinal fluke eggs. The results will be presented in our follow up report.

2. Moreover, authors may blind the egg samples and then send to 2-3 microscopic examiners and the ramen spectroscopy for evaluation of reliability, sensitivity and specificity between two techniques.

Reply: This study was conducted in a blind fashion in which the specimens were observed by two groups of observers. The first group consisted of parasitologists who prepared *O. viverrini* eggs, while the second group consisted of Raman microscopists who ran the Raman spectroscopic experiments, examined the Raman spectra, and analyzed the spectral data.

3. Authors should plan to include eggs from human subject to confirm that it can be used in the human.

Per the reviewer’s recommendation, we have run a new set of additional experiments to obtain Raman spectral data from human fecal specimens. The results are now included in the revised manuscript in the new Figures 3 and 4. The new results suggested close similarity between the Raman spectral peaks of *O. viverrini* eggs in fecal specimens of hamster and human, confirming that the methodology can be applied for detection of *O. viverrini* eggs in the human.

Again, we appreciate the effort and very helpful feedback and suggestions from the reviewers for improving the manuscript. We believe our revisions have satisfactorily addressed these concerns and that the revised manuscript presents a clearer, more scientifically complete presentation of our methodology. Again, thank you for your time and input. We look forward to your review of this revised manuscript.

Sincerely,
